# The impact of poor adult health on labor supply in the Russian Federation

**DOI:** 10.1007/s10198-016-0798-x

**Published:** 2016-04-16

**Authors:** Yevgeniy Goryakin, Marc Suhrcke

**Affiliations:** 1 Norwich Medical School, University of East Anglia, Norwich, NR4 7TJ UK; 2 Centre for Health Economics, University of York, York, UK

**Keywords:** Chronic diseases, Labor supply, Health, Russia, 9.001: I1-Health, 10: J-Labor and Demographic Economics

## Abstract

We examine the labor supply consequences of poor health in the Russian Federation, a country with exceptionally adverse adult health outcomes. In both baseline OLS models and in models with individual fixed effects, more serious ill-health events, somewhat surprisingly, generally have only weak effects on hours worked. At the same time, their effect on the extensive margin of labor supply is substantial. Moreover, when combining the effects on both the intensive and extensive margins, the effect of illness on hours worked increases considerably for a range of conditions. In addition, for most part of the age distribution, people with poor self-assessed health living in rural areas are less likely to stop working, compared to people living in cities. While there is no conclusive explanation for this finding, it could be related to the existence of certain barriers that prevent people with poor health from withdrawing from the labor force in order to take care of their health.

**Electronic supplementary material:**

The online version of this article (doi:10.1007/s10198-016-0798-x) contains supplementary material, which is available to authorized users.

## Introduction

In the past two decades, Russia has experienced a radical transformation from a socialist economy to a market economy. While creating economic opportunities for a large number of people, the process of economic disruption associated with the transition has also entailed a heavy and widely documented social and human toll for the Russian population [1]. Compared to many other Eastern European and former Soviet Union countries, Russia started out from one of the highest baseline real GDP per capita pre-transition, but subsequently suffered one of the greatest output falls. The Russian population also experienced dramatic deteriorations in a range of health outcomes [2, 3]. Out of the group of countries with comparable levels of per capita incomes, until recently Russia had one of the highest male mortality rates, and even did worse than many significantly poorer countries [4].

In contrast to most developing countries, this deterioration in health was predominantly attributable to increases in non-communicable diseases and injuries [5]. As evidenced by the large gender gap in life expectancy, it also appears that several behavioral factors, such as increased rates of smoking, excessive alcohol consumption, and mental stress, were among the principal drivers of these trends [3].

While a notable body of research has examined the determinants of Russia’s poor health [6–8], only a small amount of work has examined its consequences. For example, Abegunde et al. [9] found a small positive association between non-chronic diseases and a probability of missing days of work in Russia. However, they suggested that the weak association obtained for the chronic diseases can be explained by the fact that they conducted analysis at the household level, and that a possible improvement would be to fit the model to the individual level—the approach we have now implemented. Our paper also adds to their work in that we also consider in detail the difference in the effect of separate diseases, and that we take into account both the extensive and intensive margins of labor supply. Finally, Suhrcke et al. [4] found rather weak association between self-reported health and medically diagnosed diseases on labor supply as defined by log weekly hours. However, this result was obtained for the selected sample of those reporting only positive hours worked, ignoring the effect of health on those reporting zero hours worked. By also using a two-part model, our goal is to assess the overall effect of health on labor supply. More specifically we test the following hypotheses:

### Hypothesis 1

Poor health is expected to lead to a reduction in labor supply.

As a large proportion of the burden of chronic disease in Russia occurs among the working-age population [3], economic consequences of ill health might be considerable [10]. Yet, from a theoretical perspective, while poor health is expected to lead to a decline in productivity and therefore to lower hourly wages (and thus to individuals choosing to substitute work for leisure), the predicted effect of health on labor supply is ambiguous, because the income effect from lower wages would tend to push labor supply in the opposite direction [11]. Nevertheless, on balance, we expect the effect to be negative, given the empirical evidence from other regions [11].

### Hypothesis 2

The effect of poor health on labor supply is expected to be stronger when the effect on both the intensive and extensive margins of labor supply are taken into account.

While intuitively one might expect more serious adverse health events such as myocardial infarctions and strokes to cause bigger reductions both in productivity and in labor supply compared to other, less “shocking” conditions, this may be empirically difficult to establish, as more seriously ill people may drop out of the labor force altogether and thus report zero hours worked. If hours worked are log-transformed (as is often the case), then their regression on the illness indicator may lead to the parameter estimation only being applicable to people who did not drop out of the labor force in response to the disease. If this is the case, then the need arises to appropriately adjust for the features of the data in any analysis seeking to allow for extrapolation of the results to the whole sample, taking account of the censored nature of the data [12].

### Hypothesis 3

Higher socioeconomic status is associated with a stronger response of working to adverse health events.

A further hypothesis relates to the heterogeneity in the effect of health on labor supply, specifically on working status. Some previous literature has noted that the poor may continue working despite having serious health problems [13], simply because they cannot afford to retire or to treat their illness. In this scenario, the full economic costs of illness may be underestimated for people in lower socioeconomic status (SES). Therefore, one proposition worth examining is whether people with a higher SES are more likely to drop out of the labor force in response to adverse health events than those with lower SES.

### Hypothesis 4

Living in cities is associated with a stronger response of working status to adverse health events.

A related hypothesis is that it is not wealth per se, but rather access to appropriate medical care and social insurance mechanisms that facilitates people’s labor force exit in response to adverse health events. If this is the case, then people living in the cities may find it easier to stop working when they are ill.

### Hypothesis 5

People that are closer to retirement age are more likely to stop working in response to being in poor health.

One may expect that the potentially negative effect of poor health on currently working may be at its strongest near the retirement age, when adverse health events tend to be more serious. On the other hand, at younger ages, people may have to disregard their deteriorating health simply in order to financially sustain themselves and their families.

### Hypothesis 6

Women are more likely to stop working than men when being in poor health.

Finally, one can expect that the effect of poor health on labor supply will be stronger for women than for men across the age distribution, both because the opportunity costs of not working are usually higher for men (who tend to earn more than women), and because men are often the main family breadwinners.

In the following section, we describe the data and variables in more detail. “Empirical approach” elaborates on the specific empirical strategies required for identification of the parameters of interest. “Results” presents results and “Discussion” discusses them.

## Data

In this paper, we use data from rounds 9–18 of the Russia Longitudinal Monitoring Survey—Higher School of Economics (RLMS-HSE) dataset collected in 2000–2009 by the University of North Carolina Population Center. While we also had data available for rounds 5–8 and 19 for some covariates, it was not available for all of them. The RLMS-HSE is a household-based survey, designed to be nationally representative. Data has been collected in a repeated survey of household dwelling units since 1992, although the first part of the survey, collected until 1995, is too different to be included in this analysis. More information is on the survey website: http://www.cpc.unc.edu/projects/rlms-hse/project/sampling (last accessed in August, 2015). The sample was restricted to adults aged 18–65 years. Although pension age in Russia is in fact below 65 (i.e., 55 years for women and 60 for men), a significant proportion (i.e., about 32 %, according to our calculations) of people under the age of 65 continue working even after attaining the ‘official’ retirement age, mostly because Russian pensions (especially in earlier survey rounds) are relatively modest. Therefore, rather than excluding all respondents potentially eligible for a pension from the sample, we have included a dummy variable for those receiving a pension, either age or disability-based.

We have two dependent variables measuring labor supply: a binary indicator for current working status, and a natural log of hours worked in the last 30 days. The former variable has a value of one if a respondent says that he or she currently works, is on paid or unpaid leave, and zero otherwise. The hours worked variable was formed using the answers to the following question:

“How many hours did you actually work at your primary [secondary] place of work in the last 30 days?”

After summing reported hours worked in a primary and secondary place, we took the natural log of this variable, as the untransformed outcome variable is highly skewed.

With health being a multi-dimensional concept, we used its various definitions in our specifications. Specifically, we consider the following measures:

### Self-assessed health (SAH)

Respondents were asked to evaluate their health according to five categories, ranging from very good to very poor. Based on the responses, we created a binary variable, assigning it a value of one if respondents rated their health as being poor or very poor, and zero otherwise.

One problem with this indicator is that it may depend not only on true underlying health but also on socioeconomic status, which is also a correlate of being employed. Likewise, given that SAH usually measures underlying true health with error, and additionally assuming that this error is subject to the classical error-in-variable-assumption (i.e., not correlated with the unobserved true health variable) [12], the parameter on the relationship between SAH and labor market outcome variables may be downward-biased in the ordinary least squares (OLS) model.

### Diagnosed conditions based on self-reports

Specifically, the following indicators were derived from the answers to these questions:

-“Has a doctor ever diagnosed you as having had a stroke-blood hemorrhage in the brain?”

-“Have you ever been diagnosed with a myocardial infarction?”

-“Did a physician tell you at any time that you had diabetes or increased sugar in the blood?”

We created dummy variables with the value of one assigned to people who answered “yes”, and zero otherwise. In addition, the responses to the following questions were obtained:

-“Do you have any kind of chronic illness?”

Specifically, we defined dummies with the value of one assigned to people who answered that they had liver, lung, kidney and heart diseases. We expect parameters estimated for those diseases, as well as for diabetes, to be smaller in size than for strokes and myocardial infarctions, without, however, implying that the former diseases are not serious. Nevertheless, they may be more difficult to diagnose (in contrast to strokes and myocardial infarctions), and the resulting greater measurement error may entail a downward bias in the parameter estimates for these variables. In addition, although these chronic conditions (especially heart disease) may indeed have a debilitating effect on the ability to work, the likelihood of this happening increases with age. Since we restricted our sample to those between 18 and 65 years, we hypothesize that it is unlikely that the effect of these conditions will be as strong as for strokes and myocardial infarctions, especially given that the proportion of people self-reporting these chronic conditions is quite large (see Table 1).

**Table 1:** Summary statistics for the RLMS-HSE (1994–2009) sample

	1994	1995	1996	1998	2000	2001	2002	2003	2004	2005	2006	2007	2008	2009
Poor health	13.8 %	12.4 %	12.3 %	12.4 %	12.1 %	10.6 %	9.9 %	10.4 %	9.4 %	8.4 %	8.7 %	8.2 %	8.8 %	8.7 %
Myocardial infarction	3.1 %	3.0 %	3.3 %	3.4 %	3.6 %	3.2 %	3.1 %	3.1 %	2.8 %	2.8 %	2.5 %	2.6 %	2.6 %	2.7 %
Stroke	1.4 %	1.4 %	1.5 %	1.7 %	1.9 %	2.0 %	1.9 %	1.9 %	1.9 %	2.0 %	1.7 %	1.9 %	2.1 %	2.2 %
Diabetes	3.4 %	3.8 %	4.1 %	4.1 %	4.5 %	4.7 %	4.8 %	5.4 %	5.2 %	5.6 %	5.1 %	5.7 %	5.9 %	6.6 %
Heart disease	–	–	–	–	12.1 %	11.9 %	11.8 %	11.9 %	10.8 %	10.4 %	11.2 %	10.9 %	11.1 %	10.8 %
Liver disease	–	–	–	–	8.3 %	8.9 %	8.9 %	9.0 %	7.9 %	7.0 %	6.8 %	6.3 %	7.0 %	6.8 %
Lung disease	–	–	–	–	5.5 %	5.5 %	5.4 %	5.2 %	4.8 %	4.3 %	4.4 %	4.2 %	4.2 %	3.9 %
Kidney disease	–	–	–	–	8.4 %	9.2 %	9.1 %	8.4 %	8.6 %	7.1 %	7.4 %	6.8 %	6.9 %	6.5 %
Currently working	67.4 %	67.2 %	65.6 %	61.6 %	61.7 %	62.6 %	63.2 %	64.1 %	64.1 %	65.1 %	66.3 %	67.7 %	68.3 %	67.2 %
Age	40.6	40.6	40.6	40.7	40.7	40.4	40.5	40.3	40.2	39.9	39.7	40.0	40.1	40.5
Male	45.7 %	45.5 %	45.1 %	45.4 %	44.5 %	43.9 %	44.2 %	44.1 %	44.2 %	45.0 %	44.6 %	44.7 %	44.1 %	43.8 %
Urban	71.4 %	70.4 %	68.6 %	67.9 %	66.5 %	68.5 %	68.5 %	67.6 %	67.9 %	67.3 %	68.9 %	68.4 %	68.5 %	75.2 %

All the statistics are adjusted for the sampling weights and clustering at PSU level. All statistics reported for respondents between ages 18 and 65

We also included a set of theoretically relevant control variables in the model, i.e., age, residence (urban/rural), marital status, education, family size and number of children, wealth, household access to water, sewer, heating, hot water, as well as year and regional dummies. We also control for the number of other adults (i.e., excluding a respondent) in each household who work, as well as the number of other people with the most serious conditions-strokes, heart attacks, and with poor self-assessed health. We have also added a control for the average age of other adults in each household.

Note that although alcohol consumption and smoking may affect health, they are likely to be endogenous to work status, and therefore, we decided not to include these variables as controls. The full list of variables and their description is provided in the Appendix A1.

## Empirical approach

To start with, we are interested in estimating the parameters of the following model:

1 $$Y_{\text{it}} = {\mathbf{H}}_{\text{it}} {^{\prime} }{\varvec{\upbeta}}_{1} + {\mathbf{X}}_{\text{it}} {^{\prime} }{\varvec{\upbeta}}_{{\mathbf{2}}} + u_{\text{it}}$$

where *Y*_it_ is a binary indicator for currently working or a variable measuring log hours worked for those reporting positive hours for person *i* at time *t*; **H**_**it**_ is a vector of health dummies; **X**_**it**_ is a vector of exogenous sociodemographic controls likely to be correlated with health and labor supply, such as age, education, marital status, wealth status, urban/rural residence, household size, access to water, sewer, heating, hot water, as well as region of residence; and *u*_it_ is an error term. In the discussion that follows, we will use the term “labor supply” to describe both currently working and hours worked for those reporting positive hours worked.

In general, two major issues are likely to plague the validity of the estimated parameters of model 1. First, health may be correlated with the error term *u*_it_. For example, even conditional on including a range of covariates contained in the vector **X**_**it**_, health may still be correlated with certain unobserved determinants of *Y*_it_. Some of them, such as individual ability [14], may be time invariant, while others may change over time. Second, health may be correlated with unobserved country-wide economic shocks, which may also affect labor supply levels. To deal with these two concerns, we also estimate parameters using the following model:

2 $$Y_{\text{it}} = {\mathbf{H}}_{\text{it}} {^{\prime} }{\varvec{\upbeta}}_{1} + {\mathbf{X}}_{\text{it}} {^{\prime} }{\varvec{\upbeta}}_{{\mathbf{2}}} + \alpha_{i} + \delta_{t} + \varepsilon_{\text{it}}$$

where *α*_*i*_ is a time-invariant endowment of person *i* possibly correlated with health (e.g., ability, or level of pessimism); *δ*_*t*_ is the country-wide time effect and *ε*_it_ is the idiosyncratic error term, assumed to be independent and identically distributed (iid). We estimate model (2) by taking advantage of the panel nature of our data, i.e., by including individual-level fixed effects (IFE) as well as time effects. This allows us to control for the important source of unobserved heterogeneity in health, possibly at the expense of the loss of precision, especially if health is substantially serially correlated.

Comparing models (1) and (2), we see that we have made a restriction that the original error *u*_it_ may still contain time-varying unobserved heterogeneity, which is not controlled for in model (2). This may be viewed as a weakness of our approach, although we try to deal with it by including a large range of controls in vector **X**_**it**_. Another problem is that health may also be simultaneously determined with currently working in model (2), which neither the control variables nor individual fixed effects can address. This may happen, for example, in the context of the so-called justification hypothesis, when a person explains reduction in labor supply by reporting worse health status then they really have [15]. Having said that, the reverse effect running from labor supply to health is unlikely to be of significant concern when we measure health with diagnosed (even if self-reported) chronic or acute health conditions, especially over a short period of time. It may, however, pose a more serious problem for the SAH variable. All these issues can in principle be addressed by instrumental variable estimation. Unfortunately, no theoretically and practically convincing instruments were found in RLMS-HSE.

We model currently working status following Eq. (2) with a linear probability specification. Although this approach has some drawbacks (e.g., heteroscedastic disturbances and out-of-bound predictions), they are relatively easy to deal with, given that we are more interested in estimation than prediction [16]. It is also straightforward to estimate cluster and heteroscedasticity-robust standard errors [12]. The main benefits of this approach are the ease of interpreting coefficients, as well as computational advantages for the specifications that include individual fixed effects.

Next, we consider how poor health affects the log of the number of hours worked, also according to model (2). Although formulation (2) allows us to model the effect of poor health on the logarithm of hours worked for those reporting positive hours worked, it does not show the combined impact of health on both participation (i.e., extensive margin of labor supply) and hours worked (i.e., intensive margin). To deal with this, we treat the number of hours worked as a corner solution outcome [12], where zero represents the extensive margin side, while the variable ‘positive hours worked’ captures the intensive margin. Since the conditional expectation of hours worked is a nonlinear function of the covariates of interest, the marginal effects of health on the logarithm of hours worked can in principle be estimated by a Tobit model. However, this approach imposes the restriction that the effect of health on participation and hours worked should have the same sign. In addition, the underlying latent variable model’s errors must be normally distributed, as well as homoscedastic [12]. Instead, we recover the marginal effects using the two-part model approach [12, 17] described in detail in Appendix A2. We then compare two-part model marginal effects with those obtained when no extrapolation to the whole sample is made. Further details are available in Appendix A3.

## Results

### Descriptive statistics

We present descriptive statistics for the pooled sample of respondents between 18 years of age until 65 in Table 1. We see that the mean age of the sample participants did not fluctuate much over time, which is not surprising given the replenishment of RLMS-HSE sample in some rounds. The proportion of males has also been generally stable, fluctuating at around 44–45 % range. Unsurprisingly, the percentage of the sample population currently working appears to be strongly driven by the macroeconomic conditions, as this indicator reached its trough at the time of the 1998–1999 economic crisis.

As far as the specific health indicators are concerned, we can see that the proportion reporting their health as being poor or very poor has been on a steady decline over the observed period. This may have been due to the genuine improvement in population health, e.g., as evidenced by rising life expectancy at birth in Russia over the observation period, from 65.3 years in 2000 to 68.6 years in 2009 [18], and/or because of changing self-perception in line with the gradually improving economic situation. The proportion of working age people who have had strokes (and survived them) has been increasing until 2009. The fact that the proportion of working age people self-reporting diabetes has also been increasing suggests that an improvement in diagnostics may have played some role in this. Indeed, before 2013, the annual rate of growth of the Russian medical device market (of which the medical diagnostic segment accounted for about 43 %) was about 10–12 % [19]. As a consequence, there is some evidence of improvements in the diagnostics of specific conditions, for example chronic hepatitis between 1999 and 2009 in Russia [20]. Consequently, as the diagnosis of more serious conditions appears to have improved in more recent years, there may have been less measurement error in that period than in earlier years, and hence comparison of the prevalence of these conditions over the observed period should be made with this limitation in mind.

Finally, the proportion of patients with chronic self-reported liver, kidney, or lung disease has slightly declined over the observed period.

**Fig. 1:**
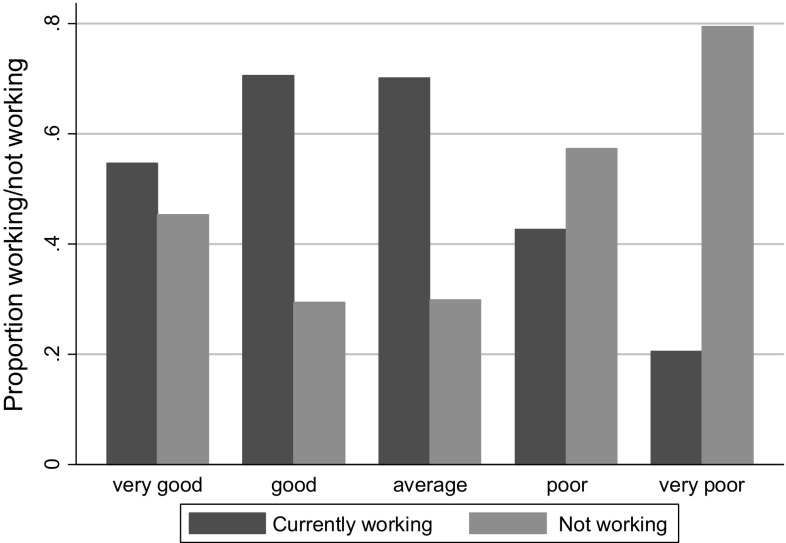
Association between self-reported health and ‘currently working’ status. Source: RLMS-HSE dataset. Sample of adults aged 18–65, inclusive

Figure 1 also shows that self-reported health is strongly related to the probability of working: among working-age adults with very poor health, about 80 % are not working. On the other hand, although the proportion of people working steadily increases with better health, the gap between those working and not working in very good health is small. However, this is not really surprising, when bearing in mind that about 63 % of the respondents who have rated their health as very good are younger than 30 years. A substantial proportion of people in this age group (about 16 %) are students, and an additional 15 % classify themselves as temporarily not working.

### Regression results

#### Baseline results

In Table 2, we examine the association between several health indicators and two individual labor market outcomes—currently working (columns 1–2), as well as the natural log of the number of hours worked in the last 30 days (columns 3–4).

**Table 2:** Association between health and individual labor market outcomes

	(1)	(2)	(3)	(4)
	Currently working	Currently working	Log hours worked (30 days)	Log hours worked (30 days)
	OLS	IFE	OLS	IFE
Poor health	−0.142*** (0.010)	−0.051*** (0.007)	−0.023** (0.010)	−0.021** (0.010)
MI	−0.038* (0.022)	−0.074** (0.030)	0.005 (0.021)	−0.019 (0.030)
Stroke	−0.077*** (0.017)	−0.128*** (0.033)	−0.003 (0.026)	0.032 (0.046)
Diabetes	0.016 (0.013)	−0.002 (0.017)	−0.005 (0.015)	−0.034* (0.018)
Heart	−0.035*** (0.010)	−0.030*** (0.006)	−0.023** (0.011)	−0.018* (0.010)
Liver	0.001 (0.010)	−0.010 (0.007)	−0.002 (0.010)	−0.003 (0.012)
Kidney	0.007 (0.009)	0.020** (0.008)	−0.000 (0.009)	0.012 (0.012)
Lung	−0.026** (0.012)	−0.003 (0.009)	−0.027** (0.013)	−0.012 (0.015)
Individual fixed effects	No	Yes	No	Yes
Observations	65,433	65,433	44,462	44,462
*R*-squared	0.247	0.053	0.043	0.005

Community cluster-robust standard errors in* parentheses*

In addition, all specifications contain round and region dummies, as well as control variables: dummies for age, pension age, being male, married, living in urban areas, having high school and university diplomas, four indicators for income status, corresponding to relevant quintiles occupied by households (adjusted for regional poverty level), dummies for household size, number of children, number of other adults in a household living with poor health; who experienced MI or strokes in the past; average age of other adults in a household; as well as controls for availability of water, cold water, sewer, and heating in the households. Sample restricted to adults between ages 18 and 65

*OLS* ordinary least squares, *IFE* individual fixed effects

*** *p* < 0.01, ** *p* < 0.05, * *p* < 0.1

For the ‘currently working’ outcome, the association is negative and significant for several “more serious” health variables: poor health, MI, stroke, heart disease in both the OLS and IFE specifications (columns 1–2). Interestingly, even with the addition of IFEs, these parameters remain significant. In the IFE model, ever having had a stroke has the largest effect on the probability of work, followed by MI and poor health. Predictably, other chronic conditions are in general more weakly related to the probability of working, or not related at all. Surprisingly, kidney disease is positively related to working in IFE model.

All control variables have the predicted signs (see Appendix A4). Thus, males, married people, those with more education, and with more wealth are all more likely to be employed than the reference groups. Family size and receiving a pension are negatively related to the probability of working. More ‘other people working’ in a household is positively related to the probability of working, while greater average age of other adults in a household is negatively related. Having other household members with poor health and strokes appears to increase the probability of work for a respondent.

The association between health and log hours worked is significant in both OLS and IFE models for only two variables—heart disease and poor health (columns 3–4). Nevertheless, we need to bear in mind that this association may be underestimated for the sample reporting positive hours only, and in our case, all those not working were excluded since we took a natural log of our outcome variable. We deal with this issue in “Extrapolating to the whole sample”.

#### Heterogeneity of health effects

An interesting issue that has rarely been dealt with in the empirical literature is how the estimated association between health and labor supply varies across a distribution of socio-demographic characteristics. In this section, we examine how one particularly popular measure of health—the SAH indicator—is related to the probability of currently working by age, wealth, residence, and gender. In this case, it is more instructive to consider the estimates for the whole sample than only for those who reported positive hours worked; therefore we chose the currently working indicator rather than log hours worked as our outcome variable. The same control variables as in Table 2 apply.

**Fig. 2:**
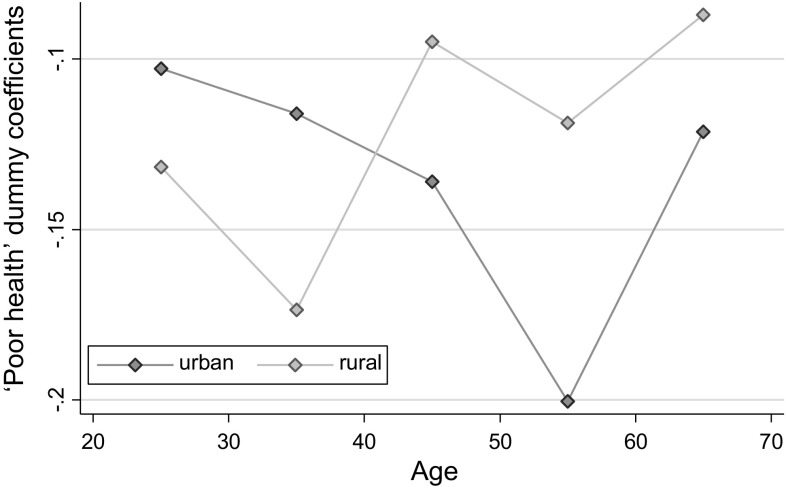
Poor health dummy regression coefficients, by age and place of residence.* Source* RLMS-HSE dataset. Sample of adults aged 18–65. The same control variables as in other specifications in Table 2 are used

In Fig. 2, we show the distribution of the coefficients from the linear probability regression of currently working dummy on poor health, stratified by age and place of residence. For the urban subsample in particular, there is some evidence that as age increases, the relationship between poor health and currently working first gets stronger, and then it weakens after retirement age. Also, as expected, for most parts of the age distribution, the effect of health on currently working for the subsample of urban dwellers is stronger.

**Fig. 3:**
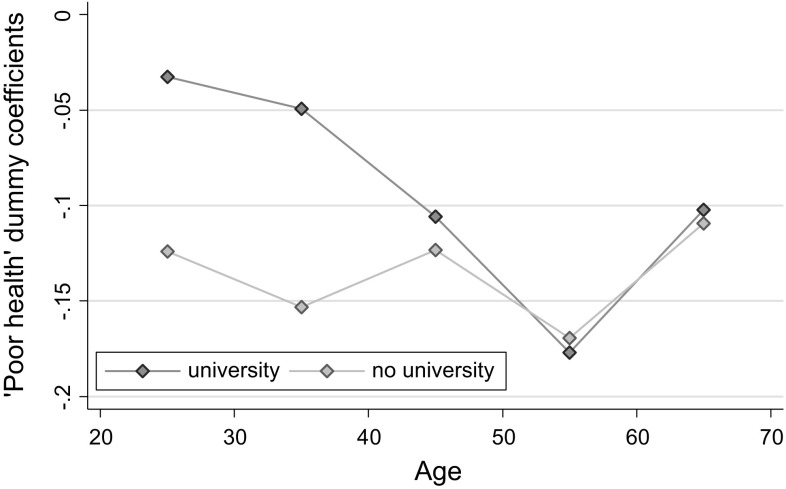
Poor health dummy regression coefficients, by age and education. * Source* RLMS-HSE dataset. Sample of adults aged 18–65. The same control variables as in other specifications in Table 2 are used

**Fig. 4:**
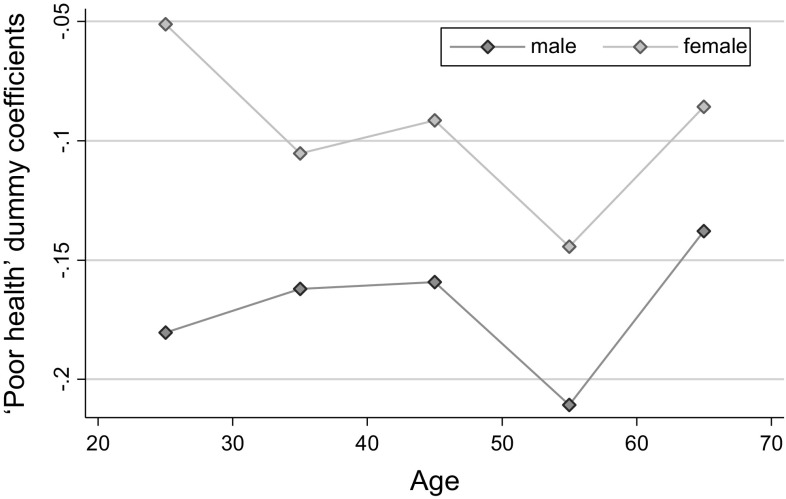
Poor health dummy regression coefficients, by age and gender. * Source* RLMS-HSE dataset. Sample of adults aged 18–65. The same control variables as in other specifications in Table 2 are used

In Fig. 3, we see that there are considerable differences in the effect of poor health by education status in younger age groups (i.e., until about 40 years), suggesting that it is people with less education (and therefore with a lower socioeconomic status) who are more likely to stop working in response to being in poor health. Finally, a somewhat surprising finding in Fig. 4 is that men are consistently more likely to stop working when they experience health problems than women.

In Table A5 in the Appendix, we show results from a more formal test of heterogeneity of health effects. To save space we only show interaction parameters between poor health and three variables of interest, i.e. being male, living in urban areas, and having university education, by two age groups. The basic message is the same as shown graphically in Figs. 2, 3, and 4. We also do the same test for two other health conditions—heart attacks and strokes. There is mostly no difference in the estimated parameters by gender or SES for these two health indicators.

#### Extrapolating to the whole sample

The results listed in “Baseline results” suggest that some health conditions may have a stronger effect on currently working than on the log of hours worked. While this is in line with our prior expectation, we should be drawing a fair comparison between the effects of health conditions on the intensive and extensive margins of labor supply. As discussed in the empirical section, we prefer to correct for this by means of a two-part model.

In Table 3, we present the two-part model results (column 1), comparing them to the parameter estimates from a log-linear model for the sub-sample of those reporting positive hours worked (column 2), with marginal effects derived using the correction described in Eq. (a7 in the Appendix).

**Table 3:** Effect of health variables on the predicted number of hours worked (last 30 days) for the whole sample

	Two-part model	Log-level model
	(1)	(2)
Poor health	−21.855*** (1.522)	−4.026** (1.792)
MI	−5.049 (3.808)	1.014 (3.938)
Stroke	−12.531*** (3.221)	−0.631 (4.734)
Diabetes	2.218 (1.969)	−0.851 (2.624)
Heart	−5.233*** (1.559)	−3.959** (1.884)
Liver	−0.129 (1.530)	−0.368 (1.774)
Kidney	1.039 (1.435)	−0.026 (1.499)
Lung	−4.169** (2.017)	−4.842** (2.170)
Observations	65,433	44,462

Community cluster-robust standard errors in* parentheses*

All specifications include contemporaneous controls, regional and round dummies (see Table 2). Sample restricted to adults between ages 18 and 65. In the first column, the dependent variable is the predicted number of hours worked for the whole sample, using formulas from Dow and Norton (2003). For participation equation, the probit model was run. In the second column, marginal effects of health on hours worked, derived from the log-level model are presented. In the first and second column, bootstrapped standard error (using 500 replications) are provided

*** p < 0.01, ** *p* < 0.05, * *p* < 0.1

The two-part model estimation shows that the largest effect on the overall number of hours worked in the last 30 days is for poor health, strokes, and heart and lung disease, which were associated with, respectively, about 22, 12, 5, and 4 fewer hours worked in the last 30 days than for the reference group. Moreover, in the log-level model with derived marginal effects according to equation a7 in the Annex (column 2, Table 3), parameter on strokes is no longer significant, and the parameter on poor health, while significant, is about five times smaller in size. The results were very similar when the normal distribution assumption of the error in the underlying log-linear model was relaxed to an i.i.d distribution assumption [21].

#### Additional checks

In Table 4, we present results from several additional checks to explore the robustness of our findings to different specifications. With column 1 serving as a baseline model (it repeats column 2 from Table 2), we can see that controlling for being satisfied with one’s life—a potential determinant of both labor market decisions and of health—makes virtually no difference to the estimated parameters (column 2). This is also true when controlling for being disabled (column 3), as well as for being disabled in the most serious, “group 1” status. The results also suggest, in line with our prior results shown in Fig. 4 as well as in Appendix A5, that for men the association between general health and current work status appears to be stronger than for women. This is also true for MI and diabetes, although the reverse is true for strokes. Finally, there was no clear pattern as to when the association was stronger: for four conditions it was stronger in 2000–2004, while for three it was stronger in 2005–2009 (Table 4).

**Table 4:** Additional checks, fixed-effects models

	(1)	(2)	(3)	(4)	(5)	(6)	(7)	(8)
	Baseline	Control for satlife	Control for disabled	Control for disabled, G1	Males	Females	2000–2004	2005–2009
Poor health	−0.051*** (0.007)	−0.042*** (0.007)	−0.042*** (0.007)	−0.045*** (0.007)	−0.073*** (0.011)	−0.035*** (0.008)	−0.030*** (0.009)	−0.065*** (0.009)
MI	−0.074** (0.030)	−0.071** (0.029)	−0.057* (0.029)	−0.058* (0.032)	−0.079** (0.034)	−0.057 (0.049)	−0.105** (0.048)	−0.032 (0.040)
Stroke	−0.128*** (0.033)	−0.121*** (0.032)	−0.110*** (0.035)	−0.121*** (0.037)	−0.123** (0.048)	−0.128*** (0.048)	−0.092 (0.060)	−0.126*** (0.041)
Diabetes	−0.002 (0.017)	−0.002 (0.017)	−0.005 (0.016)	−0.011 (0.016)	−0.060** (0.027)	0.016 (0.019)	0.016 (0.024)	−0.007 (0.022)
Heart	−0.030*** (0.006)	−0.029*** (0.006)	−0.028*** (0.006)	−0.026*** (0.006)	−0.028*** (0.010)	−0.032*** (0.008)	−0.035*** (0.008)	−0.019** (0.008)
Liver	−0.010 (0.007)	−0.010 (0.007)	−0.009 (0.007)	−0.011 (0.007)	−0.007 (0.011)	−0.012 (0.008)	−0.019** (0.009)	−0.001 (0.008)
Kidney	0.020** (0.008)	0.021** (0.008)	0.021*** (0.008)	0.020** (0.008)	0.035** (0.013)	0.013 (0.010)	0.025** (0.011)	0.010 (0.010)
Lung	−0.003 (0.009)	−0.002 (0.009)	−0.003 (0.009)	−0.002 (0.010)	−0.011 (0.015)	0.004 (0.010)	0.008 (0.012)	−0.026* (0.016)
Observations	65,433	65,177	61,979	60,805	29,764	35,669	30,119	35,314
*R*-squared	0.053	0.06	0.05	0.048	0.063	0.051	0.038	0.04

Community cluster-robust standard errors in parentheses

Row headers indicate additional controls included; or sample restriction characteristics

All models include controls for fixed effects. In addition, all specifications contain the same controls as in Table 2. G1 means “group 1”

*** p < 0.01, ** p < 0.05, * p < 0.1

Finally, we also check how work status is related to the years lived with a condition since initial diagnosis (Table 5). Specifically, we defined the date of the initial diagnosis for a particular person based on the earliest diagnosis date they mentioned for a particular condition, and then, in each round, calculated the number of years that have passed since that date, thus estimating the number of years between current round and the date of diagnosis. We then regressed current work status on this variable, which allows us to answer the following question: are more years lived with a specific condition related to the probability of working? (controlling for age and all other variables used in our previous specifications). We also assumed that when a person had never been diagnosed with a condition, that this number of years was equal to zero. This is not ideal, as strictly speaking such values should have been set to missing. However, in such a case, the sample would be very small—e.g., no more than a few hundred for some conditions. Still, as the interest is to estimate the association between labor market decisions and the accumulated disease burden as measured by the number of years that a person had been with such a condition, the zero years assumption for people without a chronic condition appears to be defendable. Note also that since in this case we consider the effect of each health variable separately, there is no need to restrict the sample to rounds 9-18 only. We were expecting to find a negative association, which was indeed the case. Results indicate that each year lived with disability reduces the probability of work by about 1 %. Each year lived after experiencing a heart attack reduces this probability by about 1.1 %. In the case of strokes, the corresponding reduction is by 2.1 %. Living with all other conditions is also significantly negatively related to the probability of working (Table 5).

**Table 5:** Effect of years since initial diagnosis on labor market outcomes

	(1)	(2)	(3)	(4)	(5)	(6)	(7)	(8)
	Currently working							
Years disabled	−0.010*** (0.002)							
Years liver		−0.005** (0.002)						
Years kidney			−0.005*** (0.002)					
Years MI				−0.011*** (0.004)				
Years stroke					−0.021*** (0.004)			
Years heart						−0.011*** (0.002)		
Years diabetes							−0.009*** (0.003)	
Years lung								−0.006** (0.003)
Observations	72,860	76,277	76,555	85,387	85,456	74,709	85,153	77,834
*R*-squared	0.063	0.072	0.073	0.078	0.078	0.072	0.079	0.074

Community cluster-robust standard errors in* parentheses*

In the top panel, outcome is current work status; in the bottom, log hours worked

All models include controls for fixed effects. In addition, all specifications contain the same controls as in Table 2

*** *p* < 0.01, ** *p* < 0.05, * *p* < 0.1

## Discussion

As expected, the negative association between health and currently working was the strongest for several conditions we had believed to be potentially more serious, e.g., indicators for poor health, MI and strokes. Interestingly, contrary to our initial expectation, one chronic disease in particular—heart disease—had a consistently strong negative association with currently working across specifications, suggesting that even though its previous diagnosis is self-reported, any downward bias resulting from potential measurement error is likely outweighed by the severity of the disease. For other chronic conditions—in the IFE specification—the association was either insignificant (liver disease, diabetes or lung disease), or even had a wrong sign (kidney disease). However, overall, four out of eight conditions had a significantly negative association with working in both the OLS and IFE specifications, with all of them being significant at less than the 5 % level in the preferred IFE model. These findings are in notable contrast to the estimated relationships between health and the log of hours worked in the last 30 days: only one condition (i.e., ‘poor health’) had a significant negative association with log hours worked in preferred IFE specification at less than the 5 % level.

In contrast, using a two-part model, we found that the effect on the predicted number of hours worked was significant for four conditions (in all of them at less than the 5 % level): self-reported health indicator, strokes, heart and lung disease, although we also found that it largely affects the extensive rather than the intensive margin. Although the effect was significant for myocardial infarctions in IFE model, it was not in the two-part model. This may be due to the selective mortality effect among the most serious cases, or potentially because “infarct” in Russia may be classified/diagnosed more broadly than “myocardial infarction” in the West.

Only scarce evidence exists on the heterogeneity of the effect of poor health on currently working across various socio-demographic characteristics. Our finding of the stronger effect of poor health on working in urban areas across most age distribution is in line with our previous hypothesis, although the reasons for this cannot be established in this paper with certainty. We speculate that this could be either because middle-aged people living in urban areas tend to have higher socioeconomic status on average (e.g., being more educated or wealthy), and therefore may find it easier to stop working and focus on getting treatment, or because they have better access to social and insurance services that allows them not to work when suffering from poor health. However, our finding that more educated people are less likely to stop working as a result of being in poor heath suggests that the latter explanation is unlikely. A more plausible explanation appears to be the availability of certain urban-specific factors that may make it easier to stop working in the cities when adverse health events occur. Finally, a possible explanation for our finding that Russian men are consistently more likely to stop working when they experience health problems than women may be that they generally tend to work in more physically demanding jobs. Alternatively, they may define “poor health” differently from women, in that for men only particularly serious conditions may be seen as a sign of “poor health”. In support of this theory, the RLMS-HSE data suggests that men are less likely to self-report poor health than women, even though they are generally unhealthier by other, more objective indicators. Nevertheless, the magnitude of the difference is quite unexpected for most age groups. One potential reason for this is that women may feel more responsible for their families and thus continue working despite being in poor health [22–24].

One potential concern in this paper is whether using a two-part model to study the effect of poor health on the logarithm of hours worked is appropriate. One may argue, for example, that what is more relevant is the effect of poor health on the potential (including missing) number of hours worked, rather than on the actual (observed) number of hours. If so, then one should determine if a selection problem exists. While similar concerns have been considered elsewhere [17, 25], we nevertheless conducted a formal statistical test for the existence of this selection problem (see the Appendix A6). Specifically, we assumed the following exclusion restriction: the number of household members could affect the number of hours worked only through their effect on the probability of working. If this assumption is valid, then the parameter on the inverse Mills ratio would identify the existence of a selection problem. We found that although the number of household members was strongly and significantly related to the probability of work, the Mills ratio parameter (lambda) was not significant.

One potential limitation of our study is that some of the variables (e.g., self-reported health or hours worked) may be measured with error, which may lead to a downward bias in the estimations, or to reduced precision. Also, the estimation may have suffered from residual endogeneity. For example, there could be some reverse feedback from labor market outcomes such as income and labor supply to health. With the data at hand, we were not able to deal with this due to the lack of good instruments. Having said that, the fact that we found significant association across a range of health indicators, including stokes and heart attacks, should alleviate this concern.

In a number of other countries, the evidence similarly suggests that poor health is an important determinant of labor market outcomes. Thus, Wolfe and Hill [26] found that in the USA, the effect of self-reported women’s health status on their work effort was stronger than on their wage rate. Walker and Thompson also found that disability, which may reflect particularly debilitating health conditions, reduced both hourly wages and labor force participation in the UK, with the effect being particularly strong on the latter [27]. With data from the Health and Retirement Study (HRS), Pelkowski and Berger found permanent health conditions to have a negative effect on labor force participation, hours worked and wages. On the other hand, temporary health problems had little to no effect [28]. Gomez and Nicolas found that in Spain, those who had suffered a serious health shock were 5 % less likely to remain employed [29]. A number of studies found that self-reported measures of health had a stronger association with labor supply than the more objective impairment and diagnosed illness indicators, which may support the justification hypothesis, which explains the association between self-reported health and labor market behavior as the result of rationalizing behavior, rather than reflecting the true effect of health [15]. On the other hand, Gertler and Gruber [30] found that labor market outcomes were more responsive to supposedly more objective functional limitations indicators than to self-reported health measures in Indonesia. Finally, a review of more recent studies, which attempted to correct for endogeneity of health [31], has concluded that health shocks significantly reduce labor force participation and work-time of the household members in low- and middle-income countries.

Overall, there were only a few broadly comparable studies conducted on data collected in Russia and the former Soviet Union region more generally. Thus, using RLMS survey data collected in 1997–2004, Abegunde et al. [9] found a significant positive association between a combined dummy for non-chronic diseases and the probability of missing days of work for heads of households. A study using data collected in ten post-Soviet countries [24], including Russia, found that poor health was associated with a 15 % lower probability of work in the community fixed-effects specification, which is comparable to the result we found in the OLS model (i.e., about 14 % lower probability of work). Finally, Suhrcke et al. [4] found that in Russia, self-assessed good health was mostly unrelated to log of hours worked per week. However, this finding could also have been due to its focus only on those who reported positive hours worked, rather than the true lack of effect. Note that we found a similarly weak association between almost all measures of health and the log of hours worked.

Frequently in the empirical literature, conclusions about the labor market consequences of poor health are based on only one particular health proxy, which may provide an incomplete and biased picture, and where such analysis is undertaken, this is frequently done for the sample of those reporting only positive hours worked. As those reporting zero hours worked are ignored, this can produce a misleading picture. By also considering the health effect on currently working in the context of a two-part model, we are more likely to accurately assess the overall effect of health on labor supply.

When considering these results, it is important to keep in mind that the effect of illness on individual or household welfare may depend not only on how labor supply responds to disease at the individual level but also on intra-household allocations of labor supply, on whether the people with an illness are in wage or salaried employment, as well as on the characteristics of the social protection system. In addition, poor health may be related to the loss of non-medical consumption that could have resulted from both greater spending on medical care as well as from income loss [32]. The welfare burden therefore may be borne by a range of players, including the individuals in poor health, other household members, their employers, or the state. For example, there might be little observed relationship between health, income, and labor supply both on individual and family levels. However, this does not necessarily mean that such health events are costless if the family has to cut back on their non-medical consumption to cover the increased medical costs, or if they have to sell off their assets in case there are no appropriate insurance mechanisms in place [13]. Alternatively, those suffering from disease(s) may have to continue working despite having poor health, which suggests an additional cost of poor health not easily captured by traditional approaches. Depending on the circumstances, this may lead to the lack of appropriate treatment, resulting in a health deterioration that could have been avoided.

Most of the existing literature on the link between health and labor market outcomes has focused on either high-income or low-income countries, paying little attention to middle-income countries such as Russia—a gap that we have addressed with the research presented in this paper. The context of Russia in particular, with its fast-paced economic reforms over the last two decades, including large-scale privatization of state-owned enterprises, provides a particularly rich ground for such research. In the future, more work on the effect of poor health in Russia on other related outcomes—including medical and non-medical consumption, intra-household allocations of labor supply, as well as individual and household-level income—will provide a useful extension to the research presented in this paper.

## Electronic supplementary material

Below is the link to the electronic supplementary material.

Supplementary material 1 (DOCX 41 kb)
