# Supplementary material for: The impact of poor adult health on labor supply in the Russian Federation
Source: Eur J Health Econ. 2016 Apr 16;18(3):361–72. doi: 10.1007/s10198-016-0798-x (PMC5355512; doi:10.1007/s10198-016-0798-x)
Supplement: Supplementary file 1 — Supplementary material 1 (DOCX 41 kb) [file 10198_2016_798_MOESM1_ESM.docx]

**Appendix**

Table A1. Variable definitions.

| **Variable** | **Description** |
| --- | --- |
| Working | Proportion reporting as working, on paid or unpaid leave at the time of the interview |
| Log hours worked | Log hours worked in the last 30 days |
| Poor health | Proportion reporting poor health |
| Heart | Have heart disease? (self-reported) |
| Kidney | Have kidney disease? (self-reported) |
| Liver | Have liver disease? (self-reported) |
| Lung | Have lung disease? (self-reported) |
| Household size | 4 dummies: 2; 3/5; 6/8; 9/13 members |
| Age | Age |
| Married | Proportion in registered marriage, or living together as partners |
| Male | Proportion of males |
| Urban | Proportion living in urban areas |
| High school diploma | Proportion having high school diploma as highest degree |
| University | Proportion having university diploma as highest degree |
| Water | Have cold water supply in house? |
| Sewer | Have sewer in house? |
| Heating | Have heating in house? |
| Hot water | Have hot water in house? |
| N of other HH members working | N/A |
| N of other HH members with stroke | N/A |
| N of other HH members with myocardial infarctions | N/A |
| N of other HH members with poor health | N/A |
| Mean age of other HH members | N/A |

*Source*: RLMS-HSE dataset. Sample restricted to people aged 18-65.

Appendix A2. Two part model estimation.

Specifically, the distribution of hours worked can be presented in the following form, describing a two-stage decision process [[19](#_ENREF_19)]:

(a1) f(y|x) =

Equation (a1) describes a conditional density function for the random variable y of interest (in our case the hours worked by the individual), which are only positive for those who work (i.e., d=1). This density is equal to the probability that a person does not participate in the labor force if hours worked are zero (y= 0), or it equals the product of the probability that a person participates in the labor force times the truncated density of y conditional on currently working and other factors X, for those with positive reported hours worked (y>0).

A natural way to model participation d in equation (a1) is by assuming that it is determined by a latent variable framework, and thus we run a probit model of the currently working dummy on all covariates of interest, using the entire sample of respondents:

(a2) *Pr[y>0| X] = Φ(X'b_1_; ε_1_)*

where y is hours worked and X is a vector of all covariates, including all health dummies. Note that y>0 implies d=1 in equation (a1), i.e. a person participating in the labor force. Using estimated parameters b_1_, we then predict currently working for the whole sample. Next, using the sample of those reporting positive hours worked, we estimate parameters in the following specification:

(a3) *Ln(y) = X'b_2_ + ε_2_,  for y>0*

This allows us to predict E[y|y>0, X] for the whole sample, using the parameter b_2_ estimated in the positive hours sample of equation a3).

Next, we estimate person-specific marginal effects of changes in health on total hours worked using the following formula, taking account of the fact that the outcome is in logs, rather than levels, as well as that the main covariates of interest are binary. In model (a4), δ^2^ is an estimator of model regression error in equation (a3), and parameter values *b_1_* and *b_2_* are estimated in models a2 and a3:

(a4)=-

We then take the average of these person-specific marginal effects for each health variable of interest, bootstrapping standard errors. Note that since the fixed effects probit estimators are inconsistent, we do not run a fixed effects two part model. However, this is not of important concern, as our main interest in this case is in comparison between the parameters estimated using censored sample (as in specification a3), and the total sample (as in specification a4), regardless of whether fixed effects are included or not.

It is also important to emphasize that the Heckman estimator is appropriate when the selection problem exists. For example, the reporting of no wage income does not necessarily mean that people work for zero wages. Rather, the wages are unobserved for them. If people who do not work are systematically different from those who do, there is a selection issue, and Heckman estimator is an appropriate way to account for missing potential wages. On the other hand, in this paper we are interested in the marginal effect of poor health on the observed hours of work. In contrast to missing wage, zero hours are the actual outcome, i.e. the result of utility maximizing behavior of individuals [[12](#_ENREF_12)]. In other words, we are not trying to solve a selection problem (which we believe does not exist), but rather we seek to account for the non-linear conditional expectation function of the outcome variable with the flexible two part model.

Appendix A3. Marginal effects estimation without extrapolation.

For comparison, we are also interested in how the marginal effects of health on hours worked differ when no extrapolation to the whole sample is made. To be able to draw an appropriate comparison, note again that our outcome variable is log-transformed, and, hence, we need to be careful in translating the parameter *β_2_* from equation (5), where the outcome is log of hours worked, into a marginal effect of health on hours worked. To put it another way, note that parameter *β_2_* estimates a semi-elasticity, but that we are interested in the effect of health on the number of *hours* worked instead. One way to do it is by estimating the difference: for each individual, and then taking the difference in mean predictions [[19](#_ENREF_19)]:

For simplicity, let us assume that model (a3) is expressed in a shortened version:

(a5)

Where variable *H_it_* is health status, for example having a certain condition, and *u_it_* is an error term. The example can be easily generalized to the case when other control variables are also included. Exponentiating both sides, we obtain:

(a6) $E(Y_{it})=\exp(\beta_{1}H_{it})\times E(\exp(u_{it}))$

From this, we can see that $E(Y_{it})$ $\neq$ $\exp(\beta_{1}H_{it})$. Since our goal is estimating , we need to make an additional correction for the error term component. Specifically, if we assume that u ~ N(0, δ^2^), then E(exp(*u_it_*)) = exp(0.5δ^2^), and therefore the marginal effect of having an illness on hours worked can be estimated as follows [[19](#_ENREF_19)]:

(a7)=

Table A4. Association between control variables and the probability of work.

|  | (1) | (2) |
| --- | --- | --- |
|  | Currently working OLS | Currently working IFE |
| Pension | -0.381*** | -0.255*** |
|  | (0.012) | (0.013) |
| Age | 0.002*** | 0.006 |
|  | (0.000) | (0.018) |
| Male | 0.035*** | - |
|  | (0.008) | - |
| Married | 0.035*** | 0.016** |
|  | (0.007) | (0.008) |
| Urban | 0.046 | -0.042*** |
|  | (0.039) | (0.013) |
| High school diploma | 0.011 | -0.031** |
|  | (0.008) | (0.013) |
| University diploma | 0.100*** | 0.172*** |
|  | (0.011) | (0.022) |
| Poverty category 2 | 0.097*** | 0.061*** |
|  | (0.014) | (0.011) |
| Poverty category 3 | 0.146*** | 0.087*** |
|  | (0.014) | (0.011) |
| Poverty category 4 | 0.193*** | 0.115*** |
|  | (0.016) | (0.012) |
| Poverty category 5 | 0.275*** | 0.157*** |
|  | (0.016) | (0.014) |
| Household size (2) | -0.047** | -0.021 |
|  | (0.019) | (0.014) |
| Household size (3-5) | -0.111*** | -0.041** |
|  | (0.025) | (0.017) |
| Household size (6-8) | -0.269*** | -0.054** |
|  | (0.041) | (0.023) |
| Household size (9-13) | -0.425*** | -0.027 |
|  | (0.055) | (0.046) |
| Number of children (1) | 0.065*** | 0.007 |
|  | (0.009) | (0.007) |
| Number of children (2) | 0.089*** | 0.002 |
|  | (0.011) | (0.011) |
| Number of children (3-8) | 0.052** | 0.016 |
|  | (0.024) | (0.019) |
| Number of other adults working in HH | 0.041*** | 0.008* |
|  | (0.009) | (0.005) |
| Mean age of other adult HH members | -0.004*** | -0.001*** |
|  | (0.000) | (0.000) |
| Number of other HH members with bad health | 0.019** | -0.006 |
|  | (0.007) | (0.005) |
| Number of other HH members with MI | 0.010 | -0.006 |
|  | (0.015) | (0.016) |
| Number of other HH members with stroke | 0.034** | 0.006 |
|  | (0.015) | (0.021) |
| Cold water available? | 0.052 | -0.016 |
|  | (0.036) | (0.019) |
| Sewers infrastructure available? | 0.021 | 0.012 |
|  | (0.033) | (0.016) |
| Heating available? | 0.014 | -0.003 |
|  | (0.019) | (0.008) |
| Hot water available? | 0.023 | -0.011 |
|  | (0.036) | (0.009) |
| Observations | 65,433 | 65,433 |
| R-squared | 0.247 | 0.053 |

*Note*: Community cluster-robust standard errors in parentheses. *** p<0.01, ** p<0.05, * p<0.1

OLS: ordinary least squares. IFE: individual fixed effects. In addition, all specifications contain round and region dummies. Sample restricted to adults between ages 18 and 65.

Table A5. Interaction parameters between poor health and three covariates, by age.

|  | (1) | (2) |
| --- | --- | --- |
|  | 18-49 | 50-65 |
|  | Poor health | |
| male X poor health | -0.104*** | -0.068*** |
|  | (0.025) | (0.025) |
| urban X poor health | -0.043 | -0.077*** |
|  | (0.029) | (0.022) |
| university X poor health | 0.096*** | 0.003 |
|  | (0.032) | (0.032) |
|  | MI | |
| male X MI | -0.084 | -0.043 |
|  | (0.075) | (0.039) |
| urban X MI | -0.006 | 0.022 |
|  | (0.080) | (0.040) |
| university X MI | -0.026 | -0.005 |
|  | (0.058) | (0.058) |
|  | Strokes | |
| male X stroke | -0.019 | -0.021 |
|  | (0.078) | (0.048) |
| urban X stroke | -0.039 | -0.052 |
|  | (0.072) | (0.042) |
| university X stroke | 0.075 | 0.102* |
|  | (0.079) | (0.052) |

*Note*: Community cluster-robust standard errors in parentheses. *** p<0.01, ** p<0.05, * p<0.1. Interaction parameters reported. In addition, all specifications contain round and region dummies, as well as control variables: dummies for age, pension age, being male, married, living in urban areas, having high school and university diplomas, 4 indicators for income status, corresponding to relevant quintiles occupied by households (adjusted for regional poverty level), dummies for household size, number of children, as well as controls for availability of water, cold water, sewer and heating in the households. Sample restricted to adults between ages 18 and 65.

Table A6. Heckman selection test

|  | (1) | (2) |
| --- | --- | --- |
|  | Log hours  worked (30 days) | Participation |
| Poor health | -0.020* | -0.436*** |
|  | (0.010) | (0.020) |
| MI | 0.005 | -0.117*** |
|  | (0.015) | (0.034) |
| Stroke | -0.009 | -0.248*** |
|  | (0.018) | (0.041) |
| Diabetes | -0.006 | 0.044* |
|  | (0.009) | (0.025) |
| Heart | -0.022*** | -0.119*** |
|  | (0.008) | (0.020) |
| Liver | -0.002 | -0.005 |
|  | (0.008) | (0.022) |
| Kidney | 0.000 | 0.023 |
|  | (0.008) | (0.022) |
| Lung | -0.028*** | -0.085*** |
|  | (0.010) | (0.026) |
| Household member number |  | -0.242*** |
|  |  | (0.010) |
|  |  |  |
| Mill's ratio lambda P-value |  | 0.919 |
| Observations | 72,315 | 72,315 |

Note: Note: Community cluster-robust standard errors in parentheses. *** p<0.01, ** p<0.05, * p<0.1

All specifications include contemporaneous controls, regional and round dummies (see table 2). Sample restricted to adults between ages 18 and 65.
